# Guardians at the Gate: Early Adversity, Neurocognitive Development, and the Role of the Pediatrician in the Era of COVID-19

**DOI:** 10.3389/fped.2021.665335

**Published:** 2021-04-14

**Authors:** Jonathan A. Berken, Nia Heard-Garris, Lauren S. Wakschlag

**Affiliations:** ^1^Department of Pediatrics, Ann & Robert H. Lurie Children's Hospital of Chicago, Chicago, IL, United States; ^2^Institute for Innovations in Developmental Sciences, Northwestern University, Chicago, IL, United States; ^3^Division of Academic General Pediatrics, Department of Pediatrics, Ann & Robert H. Lurie Children's Hospital of Chicago and Northwestern University Feinberg School of Medicine, Chicago, IL, United States; ^4^Mary Ann & J. Milburn Smith Child Health, Outreach, Research, and Evaluation Center, Stanley Manne Children's Research Institute, Ann & Robert H. Lurie Children's Hospital of Chicago, Chicago, IL, United States; ^5^Department of Medical Social Sciences, Northwestern University Feinberg School of Medicine, Chicago, IL, United States

**Keywords:** adverse child experiences, brain development, toxic stress, COVID- 19, neurocognition

## Abstract

Adverse childhood experiences (ACEs) profoundly impact neurocognitive development. Specifically, when these events occur during critical periods of brain plasticity, a time of significant synaptogenesis, neural pruning, and myelination, typical neurodevelopment can become derailed. Adverse childhood experiences promote morphological changes in neuronal microcircuitry which may lead to diminished cognitive flexibility, inattention, increased impulsivity, decreased school readiness, and disruptive behaviors. In this regard, the current COVID-19 pandemic represents an especially complex adverse experience that disturbs a child's social milieu and support network, likely interfering with brain maturation and executive function. Here, we take a neurodevelopmental approach to argue for the critical role that pediatricians must fulfill in mitigating the potentially detrimental consequences of COVID-19. We call for ACE screening and anticipatory guidance in the primary care setting, and the use of validated interventions and skills to bolster resilience, when ACEs are identified. We present a clinical workflow for the physician to proactively assess, identify, stratify, and address the severity of ACEs worsened by COVID-19. We discuss home-based activities and resources for children and adolescents to promote stress reduction, connectiveness, and self-awareness and create a more positive environment to maximize neurodevelopmental potential in the face of the ongoing pandemic.

## Commentary

Pandemics have punctuated the course of human civilization from Biblical times to the modern era. Broadly, they represent tragic milestones in history that have led to famine, war, and political upheaval. In modern times, the impact of these infectious diseases on global health has been primarily viewed in terms of their effects on adult well-being. In recent decades, however, there has been an increasing focus on the immediate and long-term risks that toxic stress, a frequent concomitant of pandemics, pose to the health of children, arguably the most vulnerable of all population groups (1, 2). To many children, the current COVID-19 pandemic represents what psychologists, pediatricians, and social service agencies refer to as an adverse childhood experience (ACE), or perhaps more fittingly, a complex of multiple adverse experiences, stemming from the restrictions imposed to mitigate the spread of the pandemic. For some children, these constraints, especially those relating to protective confinement and a lack of access to customary social outlets, are likely detrimental to physical and emotional health. For others, the challenges posed by this plague have the potential to foster greater resilience, when strong nurturing family relationships act to lessen the impact of protracted restrictions in social interaction (3). However, for those lacking such sources of support, the negative impact of the pandemic is likely to intensify, as the result of persistent isolation, stay-at-home orders, school closures, economic slowdown, parental unemployment, the death of loved ones, and separation from customary supports. Such profound changes, in the absence of compensatory factors, can result in a toxic stress response, a state of neuroendocrine dysregulation that results from prolonged exposure to adversity, threatening the future neurodevelopmental, behavioral, and emotional health of a generation of children (2, 4–6). Because the ongoing crisis has so significantly raised the stakes for our youth, especially those from low-resourced neighborhoods, it is incumbent upon pediatricians to screen for adverse conditions in a child's physical, social, and familial environment that result from or are exacerbated by this pandemic. In so doing, pediatricians become *guardians at the gate*, assuming a critical role in minimizing the negative consequences of COVID-19 on development, thus maintaining a child's potential to enjoy cognitive, emotional, and neuropsychological health throughout his or her life.

The current COVID-19 pandemic, while extraordinary, is one of many adverse experiences to which children might be exposed. Examples include chronic illness, poverty and malnutrition, racial discrimination, emotional neglect, physical maltreatment, institutionalization, parental mental illness and substance abuse, and exposure to domestic violence, experiences many of which are likely to increase in incidence as a result of the compounded stress experienced by parents and caregivers. At this juncture, the COVID-19 pandemic shows no sign of abating and, in fact, appears to be escalating. As the health crisis continues, its effects may manifest as a range of ACEs. In households at risk, job loss and food insecurity, coupled with the potential uptick in parental mental health instability and violence, may create a substrate for child abuse and neglect.

COVID-19 related ACEs may be superimposed upon adverse conditions that a child might already be experiencing, further exacerbating existing impediments to typical neurodevelopment (4–6). We know that critical periods of increased brain plasticity, including synaptogenesis, neuronal pruning, and myelination, occur from birth through the second decade of life. Deviations from normative experiences can derail typical neurodevelopment, with negative behavioral sequelae (7). This is of special importance during early childhood, a period of heightened neuroplasticity, when, depending on the nature and quality of a child's experience, a “sturdy” or “fragile” foundation for lifelong health is set via consolidation of self-regulatory skills (8).

Historically, studies of ACEs have either been retrospective and focused on adult health or prospective examining the role of ACEs as predictors of developmental dysregulation and have been almost exclusively based on observations of cognitive deficits in children raised in institutionalized settings and those suffering from malnutrition or from contact with environmental hazards (7). However, many adverse experiences are more diffuse, insidious, and range in severity. These more chronic and less extreme adverse experiences are not always recognized by clinicians. In addition, children can be exposed to several different types of adversity simultaneously, both inside and outside of the home (5). Evidence suggests that each of these forms of adversity has differential effects, and that the impact of unfavorable experiences depends on the number, timing and duration of those occurrences (5). The ACEs associated with COVID-19 are particularly likely to affect children with special needs or pre-existing mental health conditions, as well as those from immigrant backgrounds or from underserved communities, populations already in need of employment, housing, and healthcare (9).

Toxic stress in children, a correlate of ACEs, results from severe, persistent adversity often along with disruptions in parental responsiveness (6). The financial and interpersonal pressures on many families brought about by the COVID-19 pandemic, and the current constraints on play and in-person school experiences, are likely to be especially detrimental for already vulnerable children.

It is now well-established that ACEs leave both a macro- and microstructural footprint on the maturing brain, potentially through the development of a maladaptive neuroendocrine response of the hypothalamic-pituitary-adrenal axis (2). Studies have revealed that both ACEs and high levels of experienced stress are associated with morphological changes in neuronal microcircuitry and connectivity, especially in young children (10, 11). Early childhood adversity may result in significantly decreased volume of numerous brain structures, especially within the limbic system and frontal lobe, regions that continue to grow and evolve into adolescence, and that are critical to emotional regulation and executive function, as well as decreased integrity of the white matter tracts responsible for communication between these regions (11). These changes are associated with decreased cognitive flexibility and impulsivity, as well as mood and anxiety disorders. In young children, these alterations correlate with decrements in school readiness which disrupt adaptive outcomes in learning and social functioning with long-lasting impact. In older children, these brain structural variations can result in inattention, impulsivity, and hyperactivity. Thus, identifying children at risk for toxic stress due to the COVID-19 pandemic is critical, if effective intervention can be initiated to mitigate the risk for future neurodevelopmental problems (6, 9, 11, 12).

In a 2012 policy statement regarding the effects of ACEs on healthy brain development, the American Academy of Pediatrics emphasized the importance of the pediatric medical home, a family-centered partnership and community-based system where medical care for infants and children is accessible, comprehensive, and compassionate. Calling for a “broad-based, multisector commitment,” the authors entreated pediatricians to assume a leading role in “designing, implementing, evaluating, refining, and advocating for a new generation of protective interventions” (13), The current pandemic underscores the need for strengthening this focus, as it is likely that children and adolescents, particularly those with existing mental health issues and those from underserved and marginalized communities, will be disproportionately impacted (11, 12). Now, perhaps more than ever in our lifetime, children should receive special attention, most of all those who are quarantined or isolated from their parents.

Fostering a child's social, emotional, and cognitive well-being has the capacity to create a healthy environment that maximizes neurologic potential, physical health, and resilience. Intensive and frequent screening of families and communities will allow pediatricians to proactively assess for factors that might create stressful environments for the child during the COVID-19 pandemic and beyond. The effects of chronic toxic stress in childhood are likely to be cumulative and lifelong, and if not identified and addressed, can manifest as developmental delay, poor coping skills, unhealthy lifestyles, and mental illness in childhood and adolescence. In addition, adults who experience early life stress have a greater risk of heart disease, diabetes, and substance abuse (2).

A number of well-validated inventories, based on the original ACE Study (14), have proven feasible to screen for ACEs at each well-child visit (12). Among the most promising are the Traumatic Events Screening Inventory-Child Report Form Revised (TESI-CRF-R) (13, 15), a screening tool aimed at assessing children and teens from ages 6–18 in the pediatric primary care setting, the Center for Youth Wellness ACE-Questionnaire (CYW ACE-Q Child, Teen, Teen SR) (16), and the Survey of Well-being of Young Children (SWYC) (17, 18). In order for such screening tools to be broadly useful, pediatricians must be made aware of their existence and be willing to incorporate one of these ACE questionnaires into each child's clinic visit. While these inventories are helpful, they do not replace the charge of physicians to establish a strong and supportive relationship with their patients, built on trust, mutual respect, and understanding, to elicit a detailed social history from the family, and to uncover and recognize changes in the child's mood such as increased anxiety, feelings of guilt or worthlessness, anger or hostility, or anhedonia, and somatic complaints such as muscle tension, headaches, abdominal pain, anorexia, and insomnia. It is imperative, therefore, that time for conversation, including a thorough review of systems, be integrated into each visit. In addition, physicians should screen for resilience factors and the protective aspects of their young patients' environments that can be augmented to mitigate the potential harm of ACEs (3).

Pediatricians thus play an integral role in promoting optimal neurodevelopment for children in the era of COVID-19 and thereafter. They should incorporate greater screening for adverse experiences in the primary care setting in a non-intrusive fashion, beginning with a conversation with parents or guardians about ACEs, that provides anticipatory guidance about toxic stress and its effects during the pandemic. Research suggests that these discussions are well-received, with as many as 97% of parents and guardians in one study endorsing the importance of these conversations with primary care pediatricians (19). This interaction, in addition to uncovering ACEs, can emphasize the potential for children, adolescents, and their guardians to make positive behavioral changes that foster resilience in the face of COVID-19 prohibitions. In this regard, a useful preparatory module specifically aimed at the pediatric medical provider, relating to the identification and prevention of ACEs, is available through the CDC website (20). This and similar tools, such as the Safe Environment for Every Kid (SEEK) model, address the challenges of ACEs, propose ways to approach them (21), and provide pediatricians with a better understanding of the importance of psychosocial screening in their detection and prevention.

**Figure 1:**
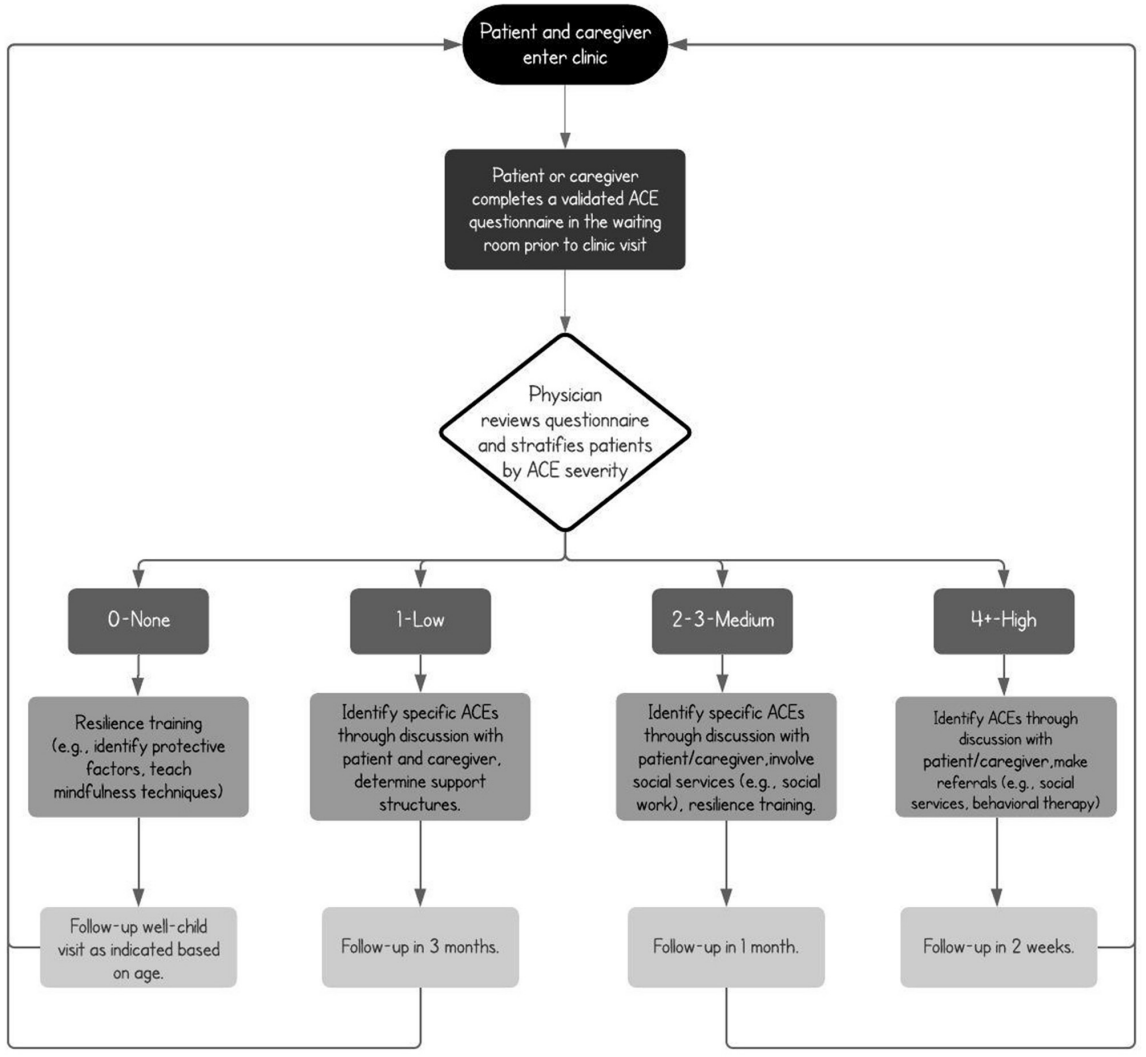
Sample clinical algorithm for ACE assessment, stratification (14), and intervention in the primary care setting, based on validated inventories. At each well-child visit, screening, detection, classification, and mitigation should be undertaken by the physician, with appropriate referrals made and close-follow up scheduled. In cases where no ACEs are found, protective factors should be determined and resilience techniques reinforced.

When ACEs are documented, pediatricians should then implement patient-specific trauma-informed care, both in the clinic and through telemedicine, to address the specific stressors affecting patients and their families, including both those specifically associated with COVID-19 and those antedating the pandemic. The pediatrician should also identify activities and resources (Table 1), such as meditation and self-reflection exercises, and supports both within the family and in the community that can be enlisted to promote social connectedness and a more nurturing environment to mitigate a child's exposure to adversity. Some of these activities are broad and have the advantage of being already routine, familiar, and comforting for children, and have been adapted for COVID-19 by various institutions including the CDC (24, 25), with versions that can be used by parents to help explain the pandemic. In addition, when ACEs are identified, pediatricians should increase the frequency of follow-up visits, involve community services such as social work and behavioral medicine, encourage parental participation in in-person and telephone parenting programs, and connect the families with home visiting and in-school-based interventions that cultivate positive parenting techniques and normative social behaviors, both in the home and the community (see Figure 1 for an example COVID-19 clinical workflow for primary care pediatricians based on previously validated inventories). It is important to note that the assessment of ACEs in the clinic depends on the training of pediatricians and allied healthcare professionals who should take it upon themselves to become familiar with the complexity of early life adversity and the many validated interventions that can be applied. To this effect, online modules related to the detection and prevention of ACEs and websites specifically focused on the unique stressors imposed by the pandemic should be reviewed by providers to increase comfort with existing resources (26).

**Table 1:** Activities and resources for COVID-19 stress mitigation.

**Activity**	**Age**	**Description**
Routine (22)	All ages	Promotes mental health and a sense of constancy at an uncertain time (e.g., morning alarms, bedtime, meal times)
Exercise (23)	All ages	Helps relieve stress and emphasizes the importance of maintaining physical health
Coloring book (24, 25)	1 and up	Promotes stress reduction and creativity
Board game (26)	3 and up	Provides opportunities for interaction with family members
Phone calls and video chats with friends and family (27, 28)	5 and up	Promotes connection with important social supports. Young children should be supervised by caregiver
Virtual trips (28)	5 and up	Provides learning experience and reduces anxiety
Simulation (28)	5 and up	Enacts stress-inducing scenarios to promote coping skills; the child can work through a hierarchy of fears about COVID-19
Yoga (29)	8 and up	Teaches relaxation techniques that help promote health and mindfulness
Conversation starters (22, 26–28)	Adolescents and teenagers	Provides prompts for difficult conversations related to the pandemic that can help relieve anxiety
Journaling (30)	Adolescents and teenagers	Promotes reflection which helps calm anxieties and identifies emotions related to COVID-19
Meditation, abdominal breathing, and muscle relaxation (31)	Adolescents and teenagers	Helps bring an accepting attitude to the moment, promotes awareness of one's body, and relieves stress

*The pediatrician can suggest and encourage patients and their caregivers to engage in age-appropriate activities to minimize anxiety and promote connectedness. Some of these activities, including the coloring book and board game, have online versions adapted to COVID-19 which can used by parents to discuss the pandemic with their children*.

At each well-child visit, parents should complete one of several short inventories, such as the Child and Youth Resilience Measure (CRYM), to help identify positive individual, familial, and community factors that promote resilience (32, 33). Providers should also learn and teach brief interventions that equip children with the coping skills necessary to navigate suboptimal environments. In doing so, they can emphasize mindfulness-based mind-body techniques that reinforce how to pay attention to and accept the moment non-judgmentally through self-awareness of one's emotions, physical sensations, and thoughts (34, 35). They can provide resilience training, cultivated through the encouragement of safe, supportive relationships, nurturing parenting styles, household routines, and the fostering of a more positive way for children to approach adverse situations (3, 26, 33–35). As the current COVID-19 pandemic threatens to leave a harmful legacy on the neurocognitive, mental, and physical health of a generation of children, pediatricians must serve as *guardians at the gate*, who, with knowledge, compassion, and patience help create environments in which children will flourish.

## Data Availability Statement

The original contributions presented in the study are included in the article/supplementary material, further inquiries can be directed to the corresponding author/s.

## Author Contributions

JB, NH-G, and LW drafted the initial manuscript, and reviewed and revised the manuscript. All authors approved the final manuscript as submitted and agree to be accountable for all aspects of the work.

## Conflict of Interest

The authors declare that the research was conducted in the absence of any commercial or financial relationships that could be construed as a potential conflict of interest.

## Abbreviations

ACE: adverse childhood experience.
